# Antioxidation and anti-inflammatory activity of Tang Bi Kang in rats with diabetic peripheral neuropathy

**DOI:** 10.1186/s12906-015-0600-0

**Published:** 2015-03-18

**Authors:** Xin-Wei Yang, Feng-Qi Liu, Jing-Jing Guo, Wei-Jie Yao, Qing-Qin Li, Tong-Hua Liu, Li-Ping Xu

**Affiliations:** School of Traditional Chinese Medicine, Capital Medical University, Beijing, 100069 China; Beijing Key Lab of TCM Collateral Disease Theory Research, Beijing, 100069 China; The Graduate School, Beijing University of Chinese Medicine, Beijing, 100029 China

**Keywords:** Tang Bi Kang, Diabetic peripheral neuropathy, Oxidative stress, Inflammation

## Abstract

**Background:**

Tang Bi Kang (TBK) is a traditional Chinese medicine granule. It has been shown to have effects on nerve conduction velocity deficits, blood-related factors and oxidative stress. This study was undertaken to evaluate proposed antioxidative and anti-inflammatory activity of Tang Bi Kang in rats with diabetic peripheral neuropathy (DPN).

**Methods:**

DPN was induced in male Wistar rats by intraperitoneal administration of streptozocin (STZ) (60 mg/kg.b.w) for 8 weeks. Fasting blood glucose (FBG) levels were measured in the blood obtained by clipping the tails of the rats. Tail-flick tests were conducted with a tail-flick analgesic meter. Motor and sensory nerve conduction velocities (MNCV and SNCV) of sciatic nerve were measured directly at two sites using a Functional Experiment System. Oxidative stress makers such as malondialdehyde (MDA), superoxide-dismutase (SOD) and glutathione peroxidase (GSH-Px), inflammatory cytokines such as interleukin (IL)-6, and tumour necrosis factor (TNF)-α were estimated. The statistical analysis of results was carried out using Student *t*-test and one-way analysis of variance (ANOVA), followed by least-significant difference post hoc with SPSS.

**Results:**

The administration of TBK for 4 weeks in DPN rats resulted in a significant decrease in FBG levels compared to untreated DPN rats. There was a significant increase in MNCV and SNCV in the DPN rats compared to untreated DPN rats. Serum level of MDA was significantly reduced while the activities of SOD and GSH-pX were significantly increased in the TBK treated DPN rats. TBK prevented DPN-induced increase in the serum levels of IL-6 and TNF-α.

**Conclusion:**

The results of this study demonstrate that the therapeutic effect of TBK on DPN rats may be associated with the antioxidative and anti-inflammatory responses.

## Background

Diabetic peripheral neuropathy (DPN) is one of the common complications of diabetes. It is manifested by MNCV and SNCV deficits as well as by increased thresholds of thermal perception [1]. The pathogenesis underlying DPN is multifactorial [2], involving the polyol pathway, hexosamine pathway, and excess/inappropriate activation of protein kinase c isoforms, accumulation of advanced glycation end products, oxidative stress and inflammation [3-5].

Hyperglycemia clearly plays a key role in the development and progression of diabetic neuropathy, and oxidative stress has been considered the final common pathway of cellular injury in hyperglycemia [6]. Recent studies show that oxidative stress and inflammation interact each other and are inseparably linked to DPN, particularly in the Nrf2–NF-κB pathway [6-9].

Nuclear factor-erythroid 2-related factor-2 (Nrf2) and nuclear factor-κB (NF-κB) are two redox regulated transcription factors, involved in oxidative stress, inflammation, cellular growth and apoptosis [10,11]. Furthermore, these two pathways are proposed to inhibit each other at transcription level via protein–protein interactions or through secondary messenger effects [12,13]. Imbalance in Nrf2–NF-κB regulation induced by hyperglycaemia is found to contribute to the pathogenesis of DPN [9]. Enhanced NF-κB activity during the hyperglycemic state is associated with excess production of proinflammatory cytokines such as IL-6, TNF-α, cyclooxygenase-2 (COX-2) and induces the generation of nitric oxide synthase (iNOS). These proteins and enzymes are mediators prerequisite for the initiation and amplification of inflammatory processes in neuronal cells [12]. Reduced Nrf2 activity results in impaired antioxidative defense, which is characterized by decline in SOD, catalase and glutathione levels. Additionally, it decreases the production of detoxifying enzymes leading to nitrosative and oxidative stress [10].

Neuroinflammation due to elevated NF-κB can activate neurons and the Schwann cells, which further augments the release of proinflammatory mediators, resulting in a vicious cycle of the inflammation. It makes the nerve fibres sensitive to painful and thermal stimulus and results in sensorimotor alterations [14,15]. The manifestations of neuroinflammation and oxidative stress can cumulatively cause the structural damage which can lead to the functional, sensorimotor and biochemical deficits which are characteristic of diabetic neuropathy [9].

Because DPN does not develop with either temporal or biochemical uniformity, its therapeutic management may benefit from a multifaceted approach that inhibits pathogenic mechanisms [9]. Recent reviews have pointed out the importance of targeting oxidative stress and inflammation in the treatment of DPN [16].

Tang Bi Kang (TBK) is a traditional Chinese medicine granule compound made from nine herbal medicines including *Astragali radix*, *Ligustri lucidi fructus*, *Cinnamomi ramulus*, *Paeoniae radix rubra*, *Spatholobi caulis*, *Scutellariae radix*, *Coptidis rhizoma*, *Hirudo* and *Corydalis rhizoma*. Studies have shown that TBK has effects on nerve conduction velocity deficiency [17] and blood factors such as endothelin-1, nitric oxide, thromboxane B2, and 6-keto-prostaglandin [18] in the STZ-induced diabetic rats. In addition, TBK was found to prevent vascular endothelial cells from oxidative stress induced by high glucose, which indirectly plays a role in protection of DPN [19].

Based on the previous studies, we hypothesized that TBK may be effective to treat DPN by reducing oxidative stress and inflammation. The aim of this study was to evaluate the therapeutical effect of TBK on DPN in rats.

## Methods

### TBK extract

TBK is made up with nine traditional Chinese medicines (Table 1). All the slices were punched from Yanjing Shuangqiao Slices Factory (Beijing, China) and were identified and authenticated by Shiyuan Jin, Honorary Professor, School of Traditional Chinese Medicine, Capital Medical University. The voucher specimen of this material has been deposited in a publicly available herbarium in School of Traditional Chinese Medicine. *Astragali radix*, *Ligustri lucidi fructus*, *Cinnamomi ramulus*, *Paeoniae radix rubra* and *Spatholobi caulis* were macerated for 1 h at room temperature with ten time volumes (v/w) of distilled water. The mixture was decocted thrice for 2 h each. The filtrates were mixed and condensed, added with alcohol to a concentration of 70%, and concentrated under reduced pressure at low temperature (40°C to 45°C) in Buchi rotavapor R-200. *Scutellariae radix*, *Coptidis rhizoma*, *Hirudo* and *Corydalis rhizoma* were macerated with six time volumes of (v/w) 70% alcohol, and reflux-extracted thrice for 1 h each, combined and dried under reduced pressure. The alcohol extract was 18.5% of total plant mass. All the extracts were stored at 0°C in airtight containers for further studies.

**Table 1:** **Recipe of TBK formulation**

**Latin name**	**Amount (g)**
*Astragali radix*	15.0
*Ligustri lucidi fructus*	10.0
*Cinnamomi ramulus*	9.0
*Paeoniae radix rubra*	9.0
*Spatholobi caulis*	15.0
*Scutellariae radix*	5.0
*Coptidis rhizoma*	3.0
*Hirudo*	1.0
*Corydalis rhizoma*	5.0
Total amount	72.0

### Animals

Animal experiments were conducted in accordance with the NIH Principles of Laboratory Animal Care and the institutional guidelines for the care and use of laboratory animals at Capital Medical University (Beijing, China). Adult male Wistar rats weighing 200 ± 20 g were obtained from the Experimental Animal Center at Capital Medical University, Beijing, China, with license number SCXK (Beijing) 2012–0001 and animal experimental ethical inspection number 2013-X-30. The rats were maintained on a 12-h/12-h light/dark cycle at 23 ± 2°C and 55 ± 10% relative humidity and allowed free access to water and standard laboratory chows.

### Induction of experimental DPN

In this experiment, a total of 80 rats were used. After overnight fasting, diabetes was induced by intraperitoneal injection of STZ dissolved in 0.1 M sodium citrate buffer (pH 4.5) at a dose of 60 mg/kg using 60 rats. On the third day, rats with FBG levels greater than 16.7 mmol/L were treated as diabetic rats and were used for further experiments. For control, 20 rats were treated with the same volume of cold citrate buffer as the nondiabetic rats.

Eight weeks later, 10 nondiabetic rats and 10 diabetic rats were randomly chosen to determine the development of DPN by measuring FBG, nociceptive threshold, and then were measured sciatic nerve conduction velocity and examined for sciatic nerve pathology. All parameters including slowed conduction velocity, increased tail-flick latency and sciatic nerve segmental demyelination and axon atrophy indicated that the model was successfully established.

### Experiment design

A total of 50 DPN rats were randomly divided into four treatment groups and a DPN model group as described in Table 2. Rats in three treatment groups were intragastrically administered with different concentrations of TBK (dissolved in 0.5% sodium carboxymethylcellulose) at 1 ml/100 g once a day for 4 weeks. Another group of DPN rats were treated with Mecobalamin (MCB). The DPN model and control groups received 0.5% sodium carboxymethylcellulose.

**Table 2:** **Experimental groups and treatments**

**Group**	**N**	**Animals**	**Treatment**
Control	10	Normal rats	0.5% sodium carboxymethylcellulose
Mod	10	DPN rats	0.5% sodium carboxymethylcellulose
MCB	10	DPN rats	0.225 mg Mecobalamin/kg b.w.
LTBK	10	DPN rats	4.28 g TBK/kg b.w.
MTBK	10	DPN rats	8.56 g TBK/kg b.w.
HTBK	10	DPN rats	17.12 g TBK/kg b.w.

### Blood glucose

FBG levels were measured biweekly in the blood obtained by clipping the tails of the rats before and after the diabetic states.

### Tail-flick test

Tail-flick latency tests were conducted before and after the diabetic state at the 4^th^, 8^th^, and 12^th^ week. The pain threshold to a thermal stimulus was assessed by tail-flick latency evoked by a noxious heat stimulus, as determined with a tail-flick analgesic meter (YLS-12A, Yi Yan, Shan Dong, China). In the tail-flick test, radiant heat was focused on the dorsal tail surface 1 cm from the tip of the tail. The cut-off time for the tail-flick reaction was set to 16 s to avoid damage to the tail.

### Sciatic nerve conduction velocity

After intragastric administration for 4 weeks, the animals were anesthetized with 10% chloral hydrate (i.p., 10 ml/kg). The stimulating and recording electrodes were placed directly under the sciatic nerve of right leg. The skin was incised and detached between the biceps femoris and semitendinous muscles to expose the sciatic nerve at the two sites, to attach electrodes. The stimulation electrode was placed at the sciatic nerve notch, and distance between the stimulation electrode and recording electrode was 6 mm. The sciatic nerve was stimulated with single square wave pluse (1.2 V in intensity, 1 ms in width) using the Functional Experiment System (BL-420 s, Taimeng, Sichuan, China). The action potential latency (L) of sciatic nerve and the distance (D) between the stimulating and recording electrodes were measured to calculate the MNCV (MNCV (m/s) = D/L). For SNCV, the site of recording was located in the sciatic notch. SNCV was calculated the same as the MNCV.

### Microscopy

After the conduction velocity test, the left sciatic nerve was isolated and cut into two segments, one segment (2 mm) was fixed in 2.5% glutaraldehyde kept at 4°C, and sent to the Electron Microscopy Center of Institute of Capital Medical University for ultrastructure observation. The other segment (1 cm) was fixed in 10% buffered formalin and processed for paraffin block preparation. Sections of about 4 μ m transverse and longitudinal thickness were cut for Hematoxylin-eosin (HE) staining.

### Immunochemistry

After the isolation of sciatic nerve, blood samples were collected, and centrifuged at 3000 rpm for 10 min to obtain serum. All serum samples were stored at −80°C.

The serum levels of inflammatory markers, including interleukin (IL)-6 and tumor necrosis factor (TNF)-α were detected using the commercial enzyme-linked immunosorbent assay (ELISA) kits (Bluegene Biotech, Shanghai, China). All experimental procedures were performed according to the manufacturer’s instructions.

### Oxidative stress

Oxidative stress was evaluated by assessing the contents of malondialdehyde (MDA), superoxide-dismutase (SOD) and glutathione peroxidase (GSH-Px). MDA content was assessed using thiobarbituric acid reactive substances assay (Jiancheng Bioengineering, Nanjing, China) by measuring the absorbance value at wavelength of 532 nm. SOD activity was assessed using the xanthine oxidase method to measure the absorbance value at 550 nm with a SOD kit (Jiancheng Bioengineering, Nanjing, China). GSH-Px activity was estimated by the analysis of glutathione in the enzymatic reaction. Reduction of GSH is catalyzed by GSH-Px in the presence of hydrogen peroxide. One unit of enzyme activity represents a decrease in GSH concentration of 1 μm/ml of serum.

### Statistical analysis

The results were presented as means ± S.E.M, statistical significance was analyzed using Student *t*-test and one-way analysis of variance (ANOVA), followed by least-significant difference post hoc with SPSS for windows version 17.0. The values were considered to be significant when P value was < 0.05.

## Results

### Assessment of the experimental DPN rat model

After 8 weeks of STZ induction, the blood glucose levels of the rats were significantly higher than those in normal rats (*P* < 0.01), indicating the rats were hyperglycemic. A significant increase in tail flick latency was observed after 8 weeks of STZ induction (*P* < 0.01), and MNCV and SNCV were significantly reduced in the STZ-treated rats compared to the normal rats (*P* < 0.01) (Table 3).

**Table 3:** **Parameters of experimental DPN rats**

**Group**	**N**	**Blood glucose (mmol/L)**	**Tail-flick latency**	**MNCV**	**SNCV**
			**(s)**	**(m/s)**	**(m/s)**
**Control**	10	5.93 ± 0.39	3.34 ± 0.52	48.96 ± 5.34	41.62 ± 5.22
**Mod**	10	31.64 ± 2.62^aa^	8.54 ± 1.39^aa^	25.18 ± 2.94^aa^	20.39 ± 2.44^aa^

Data are given as mean ± S.E.M, ^aa^ *P* < 0.01, compared to the Control group.

**Figure 1:**
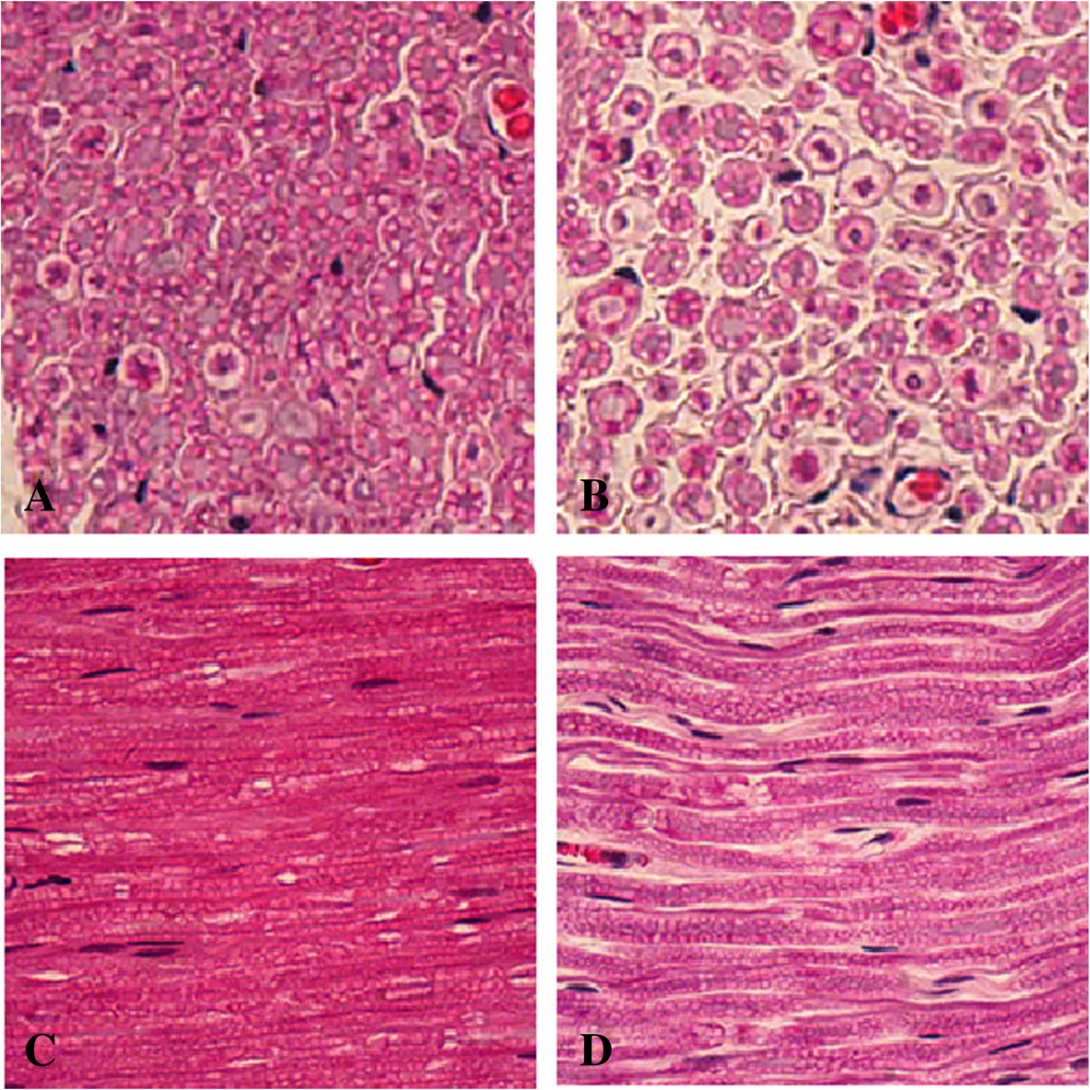
**Histological examination of hematoxylin and eosin (H&E) stained sciatic nerve.** (**A** and **C**, Control group, showing normal sciatic nerve; **B** and **D**, Mod group, showing sciatic nerve of DNP rats. Magnification, 400× ).

For the normal rats (Figure 1A and C), myelinated nerve fibers were dense, the axons were fully developed, and the thicknesses of the myelin sheaths were similar. For the STZ-treated rats (Figure 1B and D), the myelinated nerve fibers were sparsely distributed, most of myelin sheaths were not very uniform and were demyelinated. Most of the axons were swollen and some were shriveled, and the density of the myelin sheaths was not very uniform.

### Effect of TBK on FBG levels

**Figure 2:**
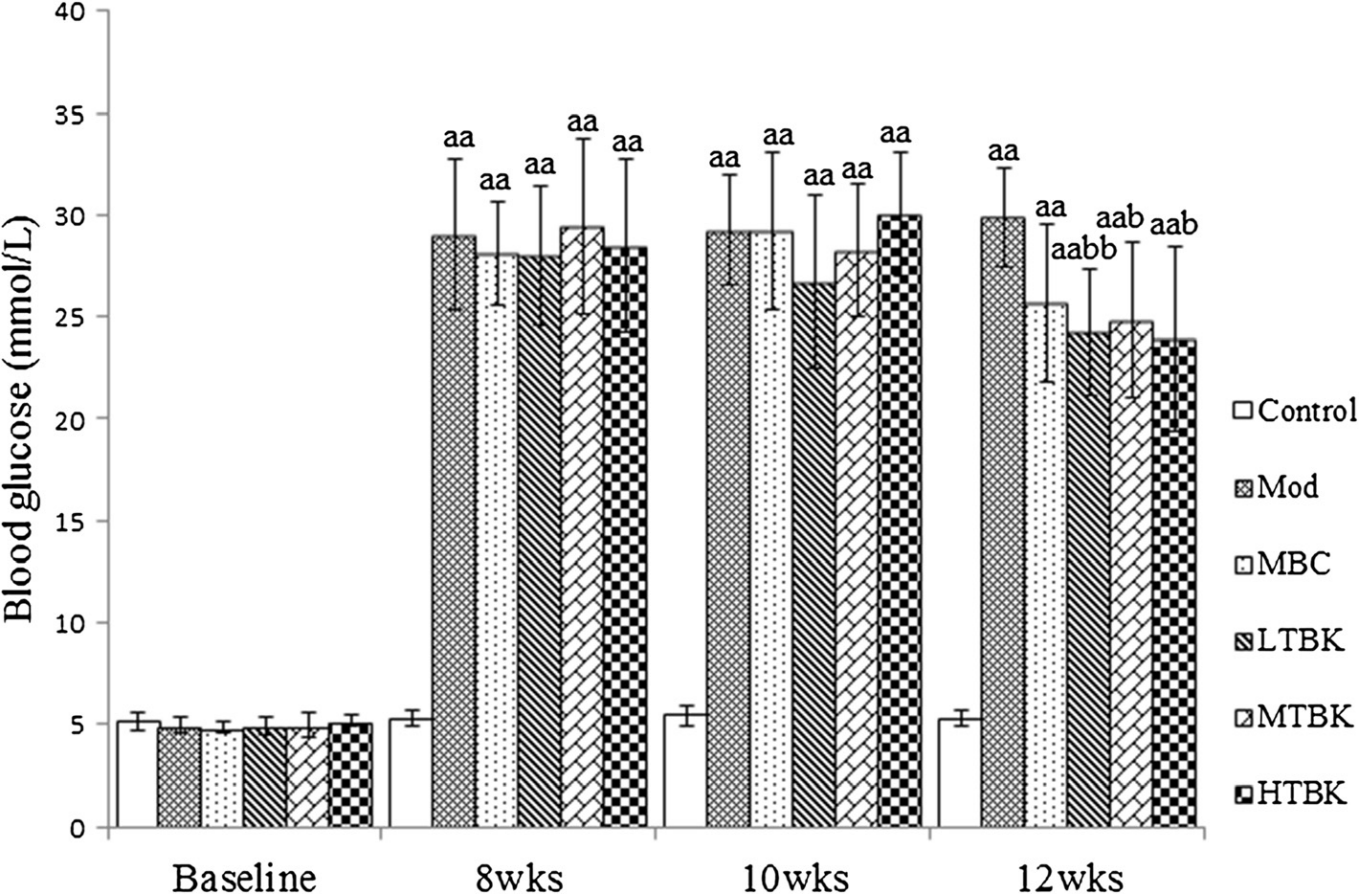
**Blood glucose levels in each group.** Each bar represents the means ± S.E.M for ten rats. ^aa^ *P* < 0.01 vs. the Control group. ^b^ *P* < 0.05, ^bb^ *P* < 0.01 vs. the Mod group.

After 8, 10, and 12 weeks of STZ induction, the FBG levels were significantly higher than those in the Control group (*P* < 0.01). For rats administered with Mecobalamin and TBK 2 for about 2 weeks (10 weeks after STZ induction), the FBG levels were not significantly different from that in the Mod group. But when administered for about 4 weeks, rats in LTBK, MTBK and HTBK groups showed a significant (*P* < 0.01, *P* < 0.05, and *P* < 0.05, respectively) decrease in the levels of blood glucose when compared with the Mod group rats (Figure 2).

### Effect of TBK on thermal perception threshold

**Figure 3:**
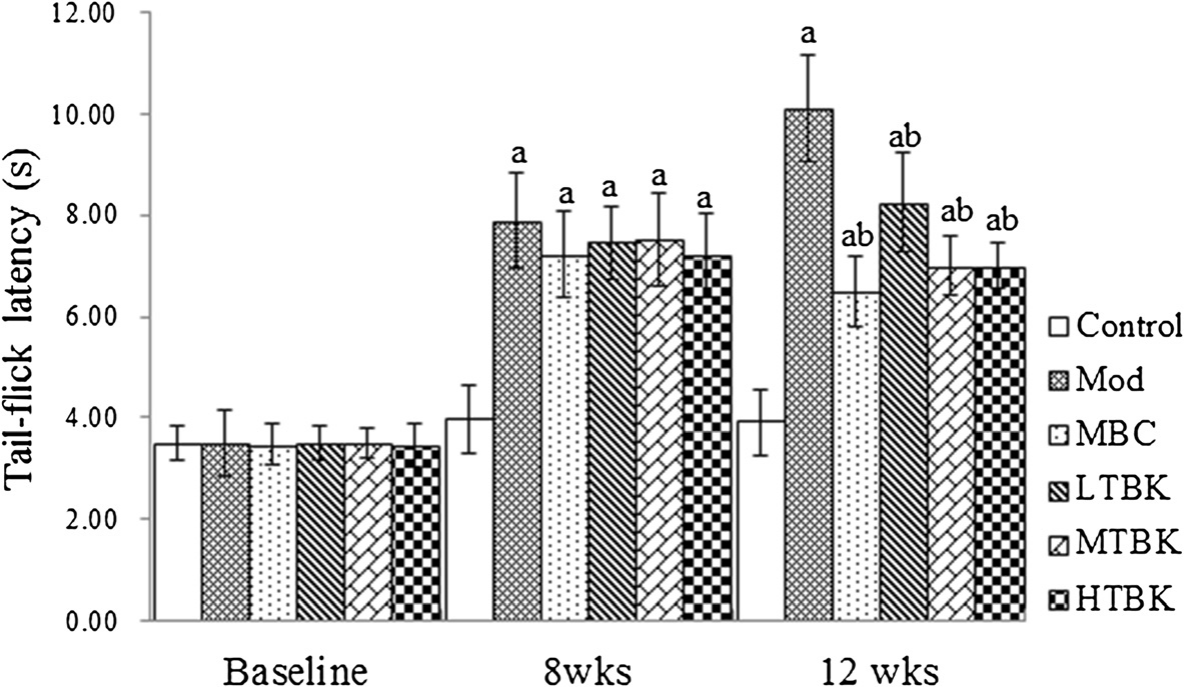
**Thermal perception thresholds in each group.** Each bar represents the mean ± S.E.M for ten rats. ^a^ *P* < 0.05 vs. the Control group. ^b^ *P* < 0.05 vs. the Mod group.

Thermal perception threshold was manifested by tail-flick latency. A significant increase in the tail-flick latency was observed after 8 weeks of STZ induction (*p* < 0.05). This deficit in tail-flick latency was significantly reversed after treatment with LTBK, MTBK and HTBK for four weeks (*p* < 0.05) (Figure 3).

### Effect of TBK on nerve conduction velocity

**Figure 4:**
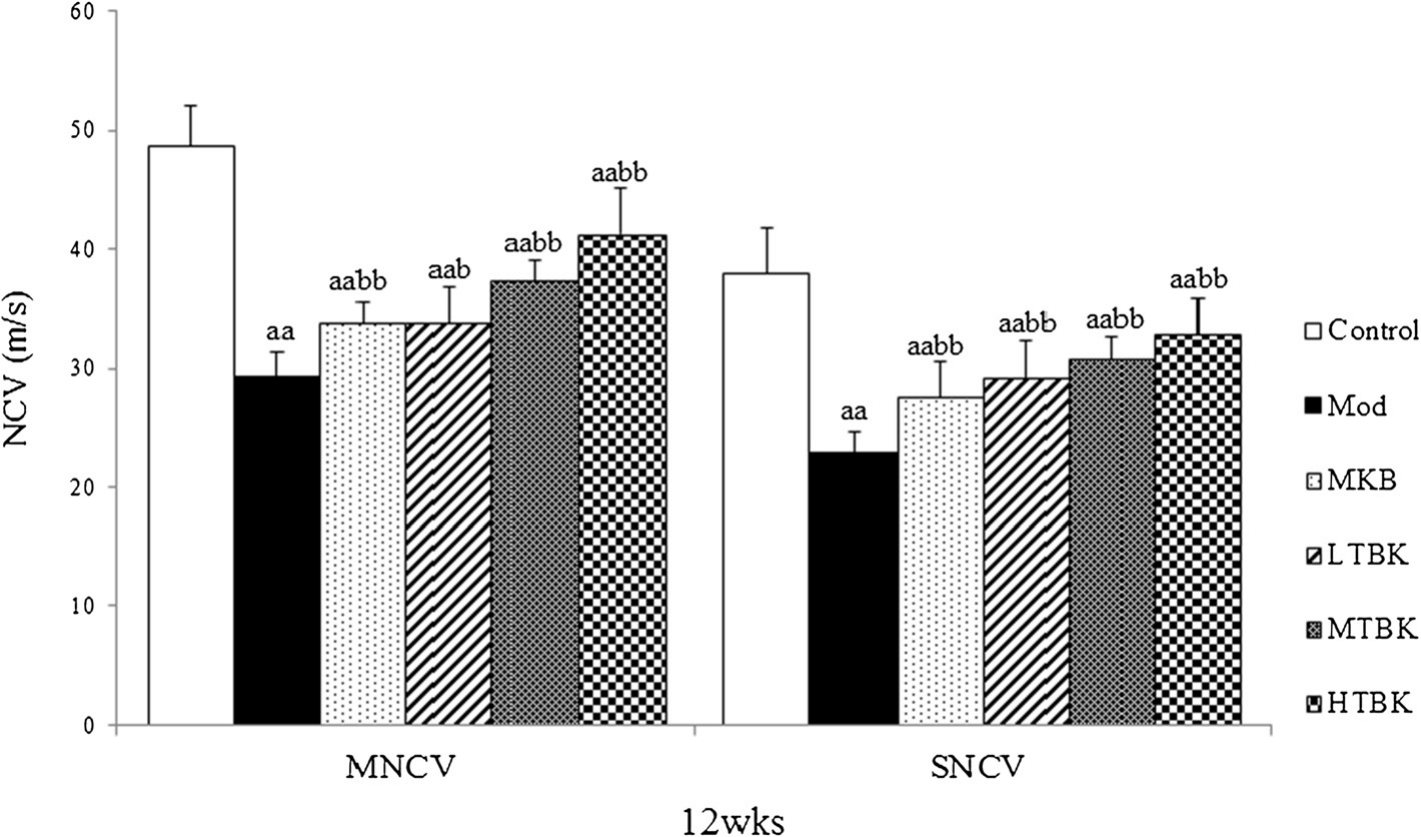
**MNCV and SNCV in Each Group.** Each bar represents the mean ± S.E.M for ten rats. ^a^ *P* < 0.05, ^aa^ *P* < 0.01 vs. the Control group. ^b^ *P* < 0.05, ^bb^ *P* < 0.01 vs. the Mod group.

Sciatic nerve MNCV and SNCV of all groups are presented in Figure 4. In Mod group, both MNCV and SNCV were significantly reduced compared with the Control group (*P* < 0.01). In LTBK, MTBK and HTBK-treated DPN rats, both MNCV and SNCV increased significantly (*P* < 0.01) compared with the Mod group rats (Figure 4).

### Effect of TBK on sciatic nerve morphology

**Figure 5:**
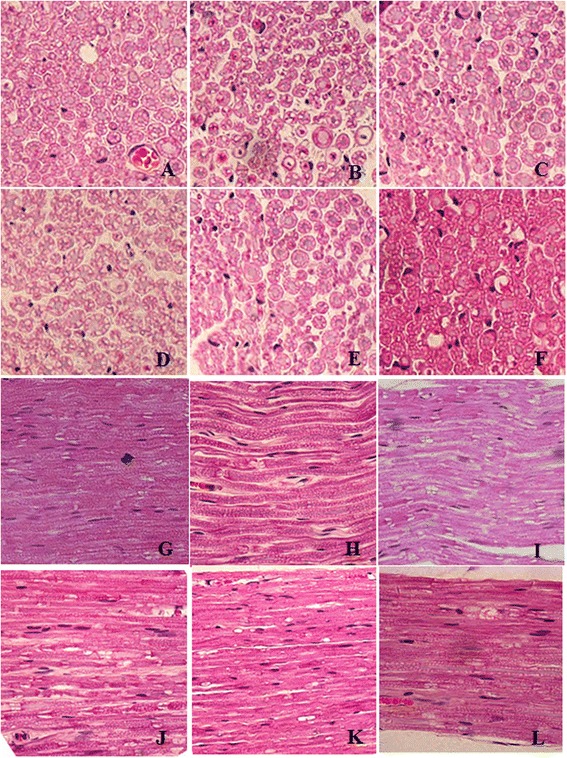
**Sciatic nerves of rats in each group.** (**A** and **H**, Control group, showing normal sciatic nerve; **B** and **I**, Mod group, showing DPN sciatic nerve; **C** and **J**, MCB group, showing sciatic nerve of DPN rats treated with Mecobalamin; **D-F** and **K-M**, LTBK, MTBK and HTBK group, showing sciatic nerve of DPN rats treated with LTBK, MTBK and HTBK, respectively. Magnification, 400 ×).

HE staining of sciatic nerve showed normal structure in control group (Figure 5A and H) and mild atrophy of axons and diffused structure of myelinated fibers at different density and size as well as demyelination in Mod group (Figure 5 B and I). The morphological change of sciatic nerve morphology in the TBK-treated rats (Figure 5 D-F and K-M) was smaller than that in Mod group.

**Figure 6:**
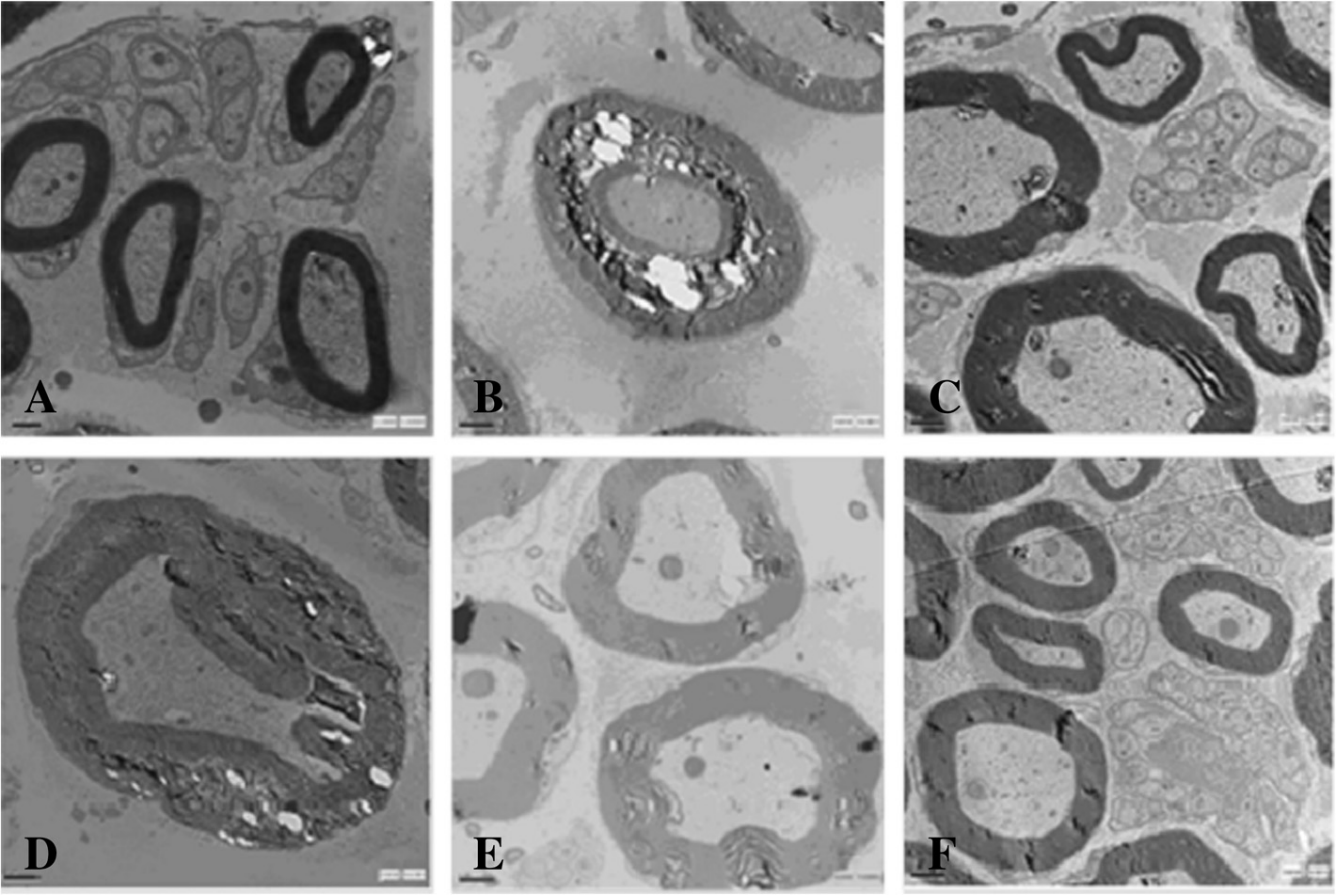
**Ultra-microstructure of sciatic nerves.** (**A**, Control group, showing normal sciatic nerve; **B**, Mod group, showing sciatic nerve of DNP rats; **C**, MCB group, showing sciatic nerve of DPN rats treated with Mecobalamin; **D-F**, LTBK, MTBK and HTBK group, showing sciatic nerve of DPN rats treated with LTBK, MTBK and HTBK, respectively. Magnification, 10000×).

For the rats in the Control group (Figure 6A), the fibers were myelinated and the myelin sheaths were dense, uniform, and arranged as concentric rings. For the rats in the Mod group (Figure 6B), the myelinated fibers were broaden, loose, and partially demyelinated; For the rats in the LTBK and MTBK groups (Figure 6D, E), the myelin sheaths were significantly broaden, and lesser demyelinated than those in the Mod group. For the rats in the HTBK group (Figure 6A), the myelin sheath structure was similar to that in the Control group.

### Effect of TBK on inflammatory cytokines

**Figure 7:**
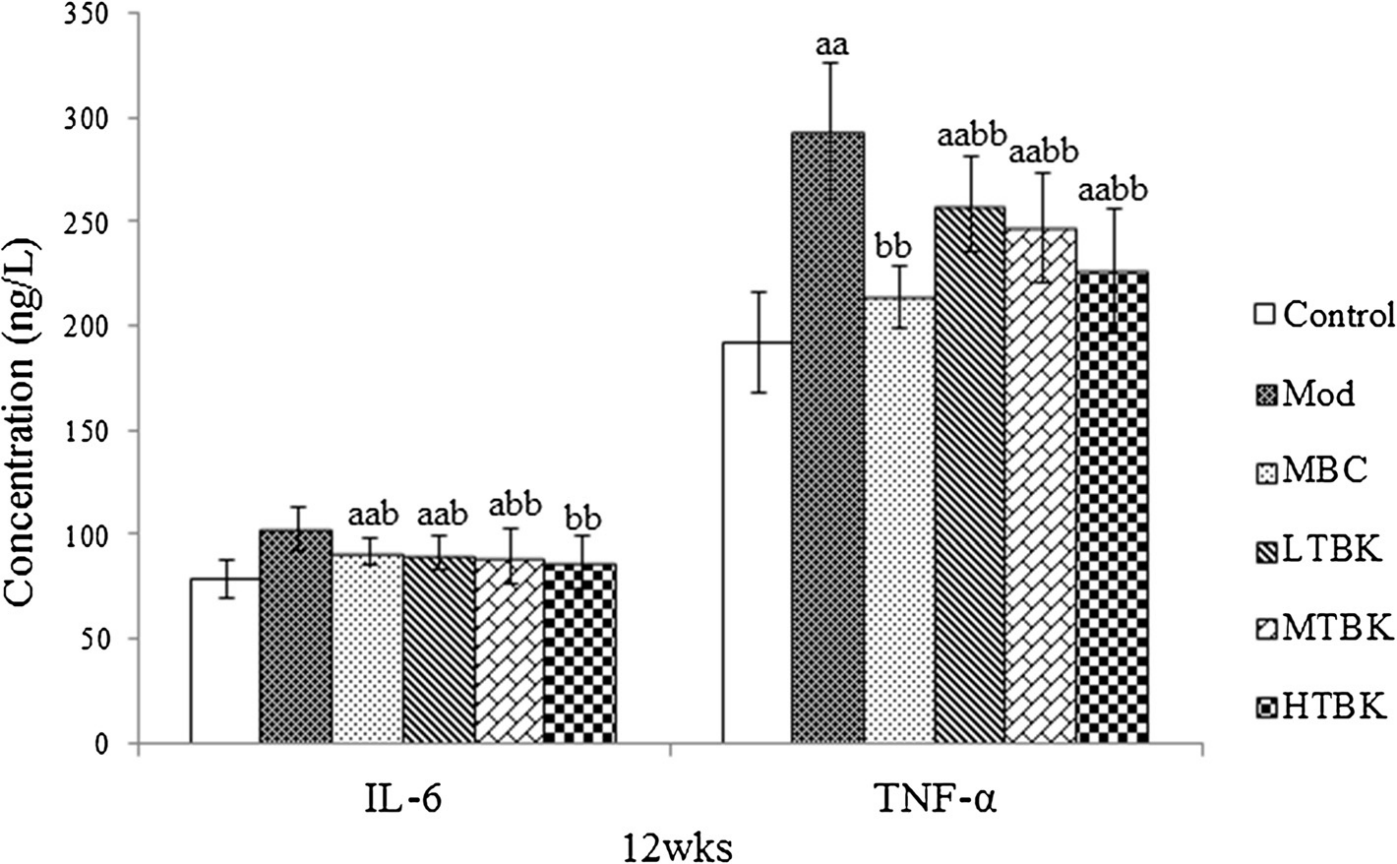
**Inflammatory Cytokine levels in each group.** Each bar represents the mean ± S.E.M for ten rats. ^a^ *P* < 0.05, ^aa^ *P* < 0.01 vs. the Control group. ^b^ *P* < 0.05, ^bb^ *P* < 0.01 vs. the Mod group.

The concentrations of pro-inflammatory cytokines TNF-α and IL-6 were significantly increased in serum of DPN rats when compared with the Control group rats (*P* < 0.01). Administration of TBK to DPN rats for 4 weeks significantly reduced TNF-αand IL-6 when compared with DPN rats (Figure 7).

### Effect of TBK on oxidative stress

**Figure 8:**
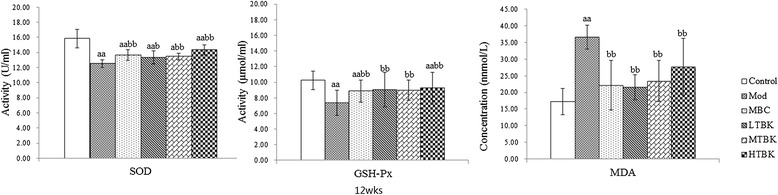
**Oxidative stress parameters in each group.** Each bar represents the mean ± S.E.M for ten rats. ^a^ *P* < 0.05, ^aa^ *P* < 0.01 vs. the Control group. ^b^ *P* < 0.05, ^bb^ *P* < 0.01 vs. the Mod group.

Lower SOD and GSH-pX were found in DPN rats compared with Control group rats (*P* < 0.01), and TBK treatment restored SOD and GSH-pX activity. Serum MDA contents were significantly increased in Mod group rats compared with those in Control group rats (*P* < 0.01), and LTBK and MTBK administration for 4 weeks decreased MDA contents (Figure 8).

## Discussion

DPN is manifested by MNCV and SNCV deficits as well as by thermal perception thresholds [20]. Nerve conduction studies can be used to quantify the nerve injury in DPN. The structural changes including axonal atrophy and loss of myelinated nerve fibers result in the reduction of MNCV and SNCV. In our study, TBK was found to reduce the axonal atrophy and demyelination, and significantly reverse the deficiency of MNCV and SNCV, and reduce the thresholds of thermal perception.

Inflammatory mediators such as cytokines have been linked to neuropathies including DPN [21,22]. Recently, increased levels of pro-inflammation cytokines IL-6 and TNF-α have been reported in diabetic rats [23,24]. TNF-α administration into the sciatic nerve of DPN patients has been shown to reduce MNCV [25]. Study showed that TNF-α- deleted diabetic mice fail to develop changes in nociceptive behavior, MNCV, and SNCV, as compared to diabetic mice expressing wild type TNF-α, and that infliximab, the TNF-α neutralizing anti-body, could recover the STZ-induced MNCV and SNCV losses, tail flick nociceptive behavior [26]. In addition, IL-6 can further exaggerate the inflammatory insults seen in diabetic nerves. Thus, these studies highlight a likely important role of TNF-α in the development of DPN. In this study, upregulation of IL-6 and TNF-α was observed in the serum of DPN rats. Our findings suggested that TBK could significantly reduce inflammation, which might be an important protective mechanism against DPN.

In the present study, oxidative stress was evaluated by measuring MDA, SOD and GSH-Px in the sciatic nerve tissue of the STZ-induced diabetic rats [27,28]. Oxidative stress has been ascribed to hyperglycemia and is regarded as primary factor in the pathogenesis of DPN [29,30]. Oxidative stress has been shown to result in metabolic dysfunction in nerve fibers, and then reduce NCV [31]. Increased oxidative stress may be associated with an overproduction of lipid peroxide and/or a significant decrease in the effectiveness of antioxidant defenses. Insufficient anti-oxidative cellular mechanisms may also be involved in nerve damage. The activities of cellular antioxidants such as the SOD and GPX may be crucial to this process [27,29]. In our study, a decreased activity of primary antioxidative enzymes such as SOD and GSH-pX in the peripheral blood of DPN rats has been demonstrated. SOD catalyzes the oxidation/reduction/conversion of superoxide radicals to molecular oxygen and hydrogen peroxide. GSH-pX catalyzes the hydrogen peroxide reduction by two molecules of glutathione, a part of reactive oxygen species defense system [32,33]. We found a significantly increased levels of MDA and decreased activity of SOD and GSH-pX in DPN rats. Our findings suggested that TBK could significantly decrease MDA and increase the antioxidative enzyme activities in DPN rats, which might be one of the protective mechanisms against DPN.

## Conclusions

The results of this study demonstrate that the therapeutic effect of TBK on DPN rats may be associated with the antioxidative and anti-inflammatory responses.

## Abbreviations

TBK: Tang Bi Kang
DPN: Diabetic peripheral neuropathy
FBG: Fasting blood glucose
MNCV: Motor nerve conduction velocity
SNCV: Sensory nerve conduction velocity
IL-6: Interleukin-6
TNF-α: Tumour necrosis factor (TNF)-α
MDA: Malondialdehyde
SOD: Superoxide-dismutase
GSH-Px: Glutathione peroxidase
ANOVA: One-way analysis of variance
Nrf2: Nuclear factor 2 related factor 2
NF-κB: Nuclear Factor-κB
COX-2: Cyclooxygenase-2
iNOS: Induced nitric oxide synthase
